# Spirometry and bronchodilator responsiveness in wheezing preschool children

**DOI:** 10.1186/1939-4551-8-S1-A161

**Published:** 2015-04-08

**Authors:** Paula Leiria-Pinto, Pedro Martins, Isabel Peralta, Elena Finelli, David Trincão, Sara Moura, Miguel Paiva, Sara Prates, Ana M Romeira, Nuno Neuparth

**Affiliations:** 1 Immunoallergy Department, Dona Estefania Hospital, Centro Hospitalar Lisboa Central, Brazil; 2 Cedoc, Chronic Diseases Research Center, NOVA Medical School / Faculdade De Ciências Médicas, Universidade Nova De Lisboa, Portugal

## Background

Recurrent episodes of wheeze are a challenging condition. Preschool wheezing children may have deficits in lung function that might lead to persistent sequelae. The aim of this study is to explore potential risk factors for reduced lung function and bronchial responsiveness to bronchodilator (BD) in children with recurrent wheezing.

## Methods

We carried out a retrospective analysis of incentive spirometry tests in recurrent wheezing children, aged 2-5 years, from our center, performed between September 2012 and March 2014. Lung function was assessed before and after 400 mg of inhaled salbutamol using a Jaeger spirometer v.4.65 (CareFusion). Wheezing symptoms, parental asthma, prematurity, passive smoking exposure, atopy and controller medication use were evaluated. Categorical frequency analysis and non-parametric tests were used.

## Results

Of 186 lung function tests performed, 158 (85%) had acceptable and reproducible criteria (children with a mean age of 4.8±0.77 years; 63.9% male). Clinical evaluation: wheezing in last year 49%; parental asthma 43%; prematurity 12%; passive smoking exposure 26%, atopy 45% and inhaled corticosteroid use 46%. We found airway obstruction in 50 (31,7%) children (FEV_1_ in 28, FEV_0,75_ in 43, FEV_0,5_ in 47 children) at baseline and in 19 (12%) after BD. A post-BD increase of 14% in FEV_t_was found in 86 tests (54%), 40 of them had basal bronchial obstruction (80% of obstruction cases). Of all risk factors evaluated, only basal bronchial obstruction was significantly associated with responsiveness to BD (p<0.001).

## Conclusions

Spirometry in preschool children with recurrent wheeze is feasible. We didn't find any association for basal reduced lung function. However, bronchial obstruction is associated with significant BD responsiveness. Therefore, in clinical practice, spirometry results may provide valuable information and could be one additional tool in wheezing management.

